# The Influence of Health Literacy on the Control of Hypothyroidism in Patients Under Levothyroxine Treatment

**DOI:** 10.1111/cen.70047

**Published:** 2025-10-06

**Authors:** Jessyka Krause Meneses, Daniella Araujo Muniz, Débora Moroto, João Roberto Maciel Martins, Carolina Castro Porto Silva Janovsky

**Affiliations:** ^1^ Endocrinology Unit, Department of Medicine, Escola Paulista de Medicina Universidade Federal de São Paulo (EPM/UNIFESP) São Paulo Brazil; ^2^ INTEGRA Clinical Research Center São Paulo Brazil

**Keywords:** adherence, health literacy, hypothyroidism, levothyroxine, patient education, self‐management, TSH

## Abstract

**Objective:**

To investigate the association between health literacy levels and biochemical control of hypothyroidism, measured by serum TSH and free thyroxine (FT4) levels, in patients receiving levothyroxine (L‐T4) therapy.

**Methods:**

We conducted a cross‐sectional study at the Thyroid Disorders Outpatient Clinic, Escola Paulista de Medicina, Universidade Federal de São Paulo (EPM/UNIFESP), between April and December 2024. The protocol was approved by the institutional ethics committee (CAAE: 76540423.5.0000.5505), and all participants provided written informed consent. Adult patients aged 18–65 years with primary hypothyroidism on levothyroxine (L‐T4) therapy were screened; 274 met eligibility criteria after exclusions. Health literacy was assessed using the Brazilian‐Portuguese Newest Vital Sign (NVS), administered face‐to‐face by trained staff. Demographic and clinical data, including comorbidities, L‐T4 dose, TSH, and FT4 levels, were extracted from electronic records. Biochemical analyses were performed using electrochemiluminescence immunoassays. Statistical analyses included ANOVA, χ² tests, and generalised linear regression, with significance set at *p* < 0.05.

**Results:**

Among 274 patients included in the final analysis, health literacy was inversely associated with serum TSH levels, and this association remained significant in fully adjusted models. A marginal trend was observed for FT4, but it did not reach statistical significance after adjustment. Patients with lower literacy scores required higher levothyroxine doses per kilogram, suggesting less efficient treatment control. No significant differences in comorbidities were observed across literacy strata.

**Conclusions:**

Limited health literacy was independently associated with poorer biochemical control of hypothyroidism, reflected by higher TSH concentrations and greater levothyroxine dose requirements. These findings reinforce health literacy as a modifiable determinant of treatment success. Incorporating literacy‐sensitive strategies—such as plain‐language counselling, teach‐back techniques, and visual aids—into routine care may help stabilise TSH, optimise levothyroxine therapy, and improve long‐term outcomes in this population.

## 1 Introduction

Hypothyroidism is one of the most prevalent endocrine disorders worldwide, affecting ≈4.6% of the population [1, 2]. Women and older adults are disproportionately affected—a pattern also observed in Brazil [1, 2]. Characterised by insufficient thyroid‐hormone production, the condition commonly leads to fatigue, weight gain and depressive mood, all of which impair quality of life [3].

Levothyroxine (L‐T4) is the standard therapy, intended to restore metabolic homoeostasis by normalising serum thyroid‐stimulating hormone (TSH) and free thyroxine (FT4) levels [1]. Nevertheless, a considerable proportion of patients continue to report symptoms or display biochemical instability. Such difficulties may stem from impaired L‐T4 absorption, drug–drug interactions, inconsistent intake or miscommunication between patients and clinicians [3, 4, 5].

Health literacy—the capacity to obtain, process and apply health‐related information—plays a pivotal role in chronic‐disease management. It encompasses cognitive and social skills that shape how individuals interpret medical advice, navigate the healthcare system and engage in self‐care. Low health literacy has been consistently linked to reduced treatment adherence, higher healthcare utilisation and poorer clinical outcomes [2, 3, 4, 5, 6].

In hypothyroidism specifically, health literacy influences patients' understanding of correct L‐T4 use and the importance of regular TSH and FT4 monitoring [7, 8]. Individuals with limited health literacy often struggle to follow complex dosing schedules or grasp the implications of laboratory results, challenges that are aggravated by low educational attainment and restricted access to reliable information [9, 10, 11, 12]. Personalised educational interventions have shown promise in improving health literacy and, consequently, clinical outcomes—including in hypothyroidism [11, 12, 13].

Given the high prevalence of hypothyroidism and the central role of L‐T4 adherence in achieving biochemical control, clarifying the impact of health literacy is especially relevant in countries such as Brazil, where socioeconomic disparities and limited access to health information persist. Despite its clinical importance, this association remains underexplored in developing‐country settings and within public healthcare systems.

Therefore, this study aims to evaluate whether health literacy independently influences biochemical control of hypothyroidism—measured by TSH and FT4 levels—in patients receiving L‐T4 therapy within a real‐world, public healthcare setting in Brazil.

## 2 Methods

### 2.1 Study Design and Ethical Approval

A cross‐sectional study was conducted at the Thyroid Disorders Outpatient Clinic, Escola Paulista de Medicina, Universidade Federal de São Paulo (EPM/UNIFESP) from April to December 2024. The protocol was approved by the Institutional Ethics Committee (CAAE: 76540423.5.0000.5505) and complied with the Declaration of Helsinki. All participants provided written informed consent (Termo de Consentimento Livre e Esclarecido).

### 2.2 Participants and Sample Size

**Figure 1:**
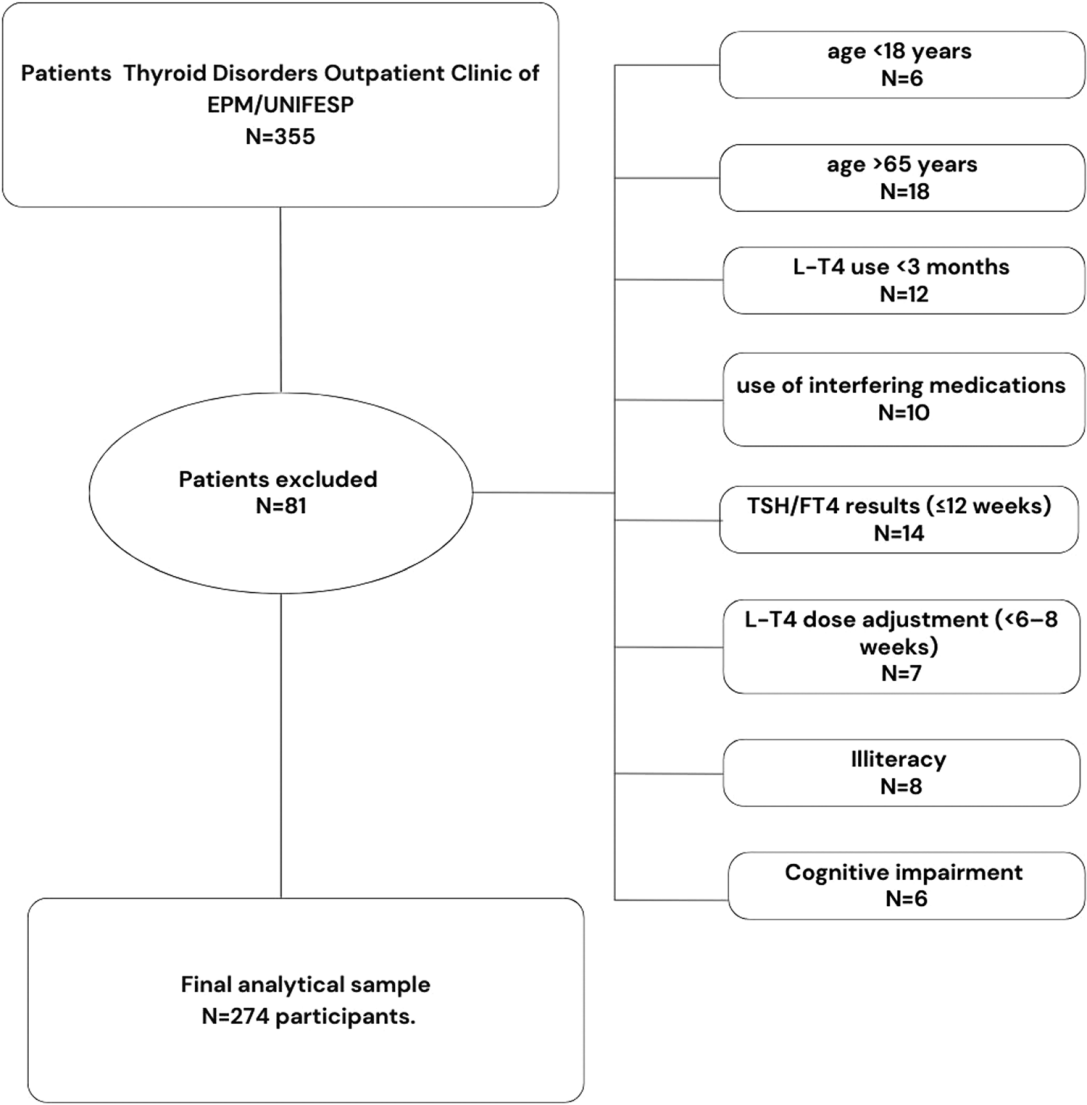
Flowchart of patient selection. Of 355 patients initially identified at the Thyroid Disorders Outpatient Clinic of EPM/UNIFESP, 81 were excluded due to predefined eligibility and data quality criteria: age < 18 years (*n* = 6), age > 65 years (*n* = 18), intermittent levothyroxine (L‐T4) use < 3 months (*n* = 12), use of interfering medications (*n* = 10), absence or out‐of‐window TSH/FT4 results ( ≤ 12 weeks) (*n* = 14), recent L‐T4 dose adjustment ( < 6–8 weeks) (*n* = 7), illiteracy (*n* = 8), and documented cognitive impairment (*n* = 6). The final analytical sample comprised 274 participants.

A single‐proportion formula for population‐based studies was used to calculate the sample size. To account for a 10% nonresponse rate, the final sample size was determined to be 355 patients. These adult patients with primary hypothyroidism receiving levothyroxine (L‐T4) were initially identified at the Thyroid Disorders Outpatient Clinic of EPM/UNIFESP. Of these, 81 were excluded based on predefined eligibility and data quality criteria (Figure 1). The final analytical sample therefore comprised 274 participants.

### 2.3 Inclusion Criteria

- Age 18–65 years.
- Primary hypothyroidism diagnosed ≥ 6 months earlier (clinical or subclinical).
- Continuous levothyroxine (L‐T4) therapy for ≥ 3 months.
- Recent TSH and free T4 (FT4) results ( ≤ 3 months old) available in the electronic record.

### 2.4 Exclusion Criteria

- Illiteracy or diagnosed cognitive disorder.
- Current use of agents that alter thyroid function or L‐T4 kinetics (e.g., amiodarone, biotin, carbamazepine, carbidopa, furosemide, haloperidol, heparin, levodopa, lithium, metoclopramide, phenytoin, propranolol, primidone, rifampin, systemic steroids, and valproic acid).

### 2.5 Data Collection

After consent, the Brazilian‐Portuguese Newest Vital Sign (NVS) questionnaire was administered by the same trained staff in face‐to‐face interviews. The NVS is a brief six‐item instrument that evaluates functional health literacy using a nutrition label as stimulus material. It requires participants to apply reading comprehension and numeracy skills to answer questions on serving size, calories, nutrient content, percentages, and allergy information. Each correct answer scores one point, yielding a total between 0 and 6. Scores of 0–2 indicate a high likelihood of limited health literacy, 3–4 suggest the possibility of limited literacy, and 5–6 almost always reflect adequate literacy. The instrument has been validated internationally and adapted for use in Brazilian Portuguese by Carthery‐Goulart et al. (2009), demonstrating good psychometric performance in outpatient populations. It typically requires 3–5 min to administer, making it feasible in routine clinical care. Although it provides a reliable measure of general health literacy, it does not directly capture disease‐specific behaviours related to levothyroxine therapy (e.g., fasting intake, timing of ingestion, or drug–nutrient interactions), which was considered in the interpretation of our findings. Demographic and clinical data—age, sex, education, treatment duration, comorbidities (obesity, dyslipidaemia, hypertension, type 2 diabetes, prior stroke or myocardial infarction)—were extracted from electronic records, along with the latest serum TSH and FT4 results (drawn ≤ 12 weeks earlier). L‐T4 dose (µg kg⁻¹ day⁻¹) was calculated from the current prescription and body weight.

Demographic and clinical data—age, sex, education, treatment duration, comorbidities (obesity, dyslipidaemia, hypertension, type 2 diabetes, prior stroke or myocardial infarction)—were extracted from electronic records, along with the latest serum TSH and FT4 results (drawn ≤ 12 weeks earlier). L‐T4 dose (µg kg⁻¹ day⁻¹) was calculated from the current prescription and body weight.

### 2.6 Laboratory Methods

#### 2.6.1 Biochemical Measurements

Serum TSH and free thyroxine (FT4) concentrations were measured in the hospital's central laboratory using an electrochemiluminescence immunoassay (ECLIA) on a Cobas e601 analyser (Roche Diagnostics, Mannheim, Germany).

‐ TSH: Analytical sensitivity (Limit of Detection) of 0.005 mIU/L, with an inter‐assay coefficient of variation (CV) < 5%. Reference interval for euthyroid adults: 0.4–4.0 mIU/L.

‐ FT4: Analytical sensitivity (Limit of Detection) of 0.5 pmol/L, with a measuring range from 0.3 to 100 pmol/L (0.093–7.77 ng/dL). The inter‐assay CV ranged from 1.3% to 4.5%, depending on the concentration. The reference interval for euthyroid adults was 12–22 pmol/L (0.93–1.7 ng/dL), according to the manufacturer's specifications.

Quality control followed CLSI standards, and the laboratory participates in the national external‐proficiency programme. All blood samples were collected in the morning during routine outpatient visits to minimise the impact of TSH circadian variation.

### 2.7 Data Management

All questionnaire and laboratory data were double‐entered into REDCap® and verified for consistency.

### 2.8 Statistical Analysis

Continuous variables are reported as mean ± SD (when normally distributed) or median (IQR); categorical variables as counts (percentages). Normality was assessed by skewness and kurtosis (–2 to +2 acceptable). Age and treatment duration were normally distributed; TSH and FT4 were log‐transformed for analysis. Group differences across NVS strata were tested with one‐way ANOVA (continuous) or χ² (categorical).

Associations between health literacy (independent variable) and biochemical/therapeutic outcomes (TSH, FT4, and L‐T4 dose) were evaluated using generalised linear regression models. For TSH and FT4, models were adjusted sequentially for age, sex, duration of diagnosis, as well as comorbidities (dyslipidaemia, diabetes, hypertension). For the outcome L‐T4 dose per kg, obesity was not included as a covariate to avoid over‐adjustment. Univariate and fully adjusted multivariable models are reported. Model performance was assessed by Akaike (AIC) and Bayesian (BIC) information criteria and log‐likelihood statistics. All analyses were performed with IBM SPSS Statistics v30, and a two‐sided *p* < 0.05 was considered statistically significant.

Group differences across NVS strata were tested with one‐way ANOVA (continuous) or χ² (categorical). Associations between health literacy (independent variable) and biochemical/therapeutic outcomes (TSH, FT4, L‐T4 dose) were evaluated with generalised linear regression:

**Model 1:** adjusted for age, sex, and log‐diagnosis duration. **Model 2:** additionally adjusted for comorbidities, which were entered as categorical variables (yes/no) and included obesity, dyslipidaemia, hypertension, and type 2 diabetes. Stroke and myocardial infarction were described in the sample but not included in the multivariable model due to low prevalence.

## 3 Results

**Figure 2:**
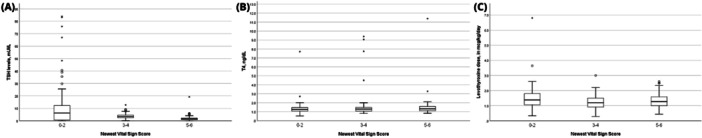
(A) Distribution of serum TSH levels (mIU/L), (B) free T4 concentrations (ng/dL), and (C) levothyroxine dose (µg/kg/day) according to health literacy levels (Newest Vital Sign score categories: 0–2, 3–4, 5–6). Boxplots show medians, interquartile ranges, and outliers. Significant differences were observed for TSH (*p* < 0.001) and levothyroxine dose (*p* = 0.011), but not for FT4 (*p* = 0.166).

A total of 274 participants were included in the final analysis. The majority were female (80.7%), with a mean age of 53.98 years (SD = 16.09). Stratification by Newest Vital Sign (NVS) scores revealed that 33.9% of participants scored 0–2, 32.5% scored 3–4, and 33.6% scored 5–6 (Table 1, Figure 2).

**Table 1:** Sample characteristics according to health literacy levels. Continuous variables are presented as mean ± standard deviation (SD) if normally distributed, or median (interquartile range, IQR) if skewed (TSH, FT4).

	Total (*n* = 274)		The Newest Vital Sign Score						*p* value
			0–2 (*n* = 93, 33.9%)		3–4 (*n* = 89, 32.5%)		5–6 (*n* = 92, 33.6%)		
Age, in years (mean, SD)	53.98	16.09	53.62a	16.49	55.27a	16.19	53.09a	15.66	0.639
TSH, in mIU/L (median, iQR)	2.67	1.24–5.27	6.24a	0.64–12.56	3.43b	2.47–4.50	1.60c	0.85–2.18	< 0.001*
T4, in ng/dL (median, iQR)	1.30	1.09–1.48	1.25a	1.07–1.46	1.29a	1.06–1.47	1.32a	1.11–1.59	0.166*
Levothyroxine dosage, in µg/kg/day (median, iQR)	1.30	1.09–1.48	1.38a	1.06–1.81	1.20b	0.91–1.51	1.27ab	1.00–1.59	0.011*
Duration of diagnosis, in years (median, iQR)	11.00	6.00–19.25	12.00a	8.00–21.50	11.00a	6.00–18.00	10.50a	6.00–19.75	0.244
	N	%	N	%	N	%	N	%	
Sex									0.777
Male	53	19.3%	16	17.2%	19	21.3%	18	19.6%	
Female	221	80.7%	77	82.8%	70	78.7%	74	80.4%	
Level of Education									0.668
Missing	178	65.0%	60	64.5%	56	62.9%	62	67.4%	
No schooling	15	5.5%	7	7.5%	5	5.6%	3	3.3%	
Elementary/Middle/High school	66	24.1%	21	22.6%	25	28.1%	20	21.7%	
Some college or higher	15	5.5%	5	5.4%	3	3.4%	7	7.6%	
Obesity									0.647
No	212	77.4%	69	74.2%	71	79.8%	72	78.3%	
Yes	62	22.6%	24	25.8%	18	20.2%	20	21.7%	
Dyslipidaemia									0.615
No	180	65.7%	60	64.5%	56	62.9%	64	69.6%	
Yes	94	34.3%	33	35.5%	33	37.1%	28	30.4%	
Hypertension									0.234
No	146	53.3%	49	52.7%	42	47.2%	55	59.8%	
Yes	128	46.7%	44	47.3%	47	52.8%	37	40.2%	
DM2									0.657
No	228	83.2%	78	83.9%	76	85.4%	74	80.4%	
Yes	46	16.8%	15	16.1%	13	14.6%	18	19.6%	
Prediabetes									0.216
No	240	87.6%	80	86.0%	75	84.3%	85	92.4%	
Yes	34	12.4%	13	14.0%	14	15.7%	7	7.6%	
Depression									0.183
No	234	85.4%	75	80.6%	76	85.4%	83	90.2%	
Yes	40	14.6%	18	19.4%	13	14.6%	9	9.8%	
AMI									0.695
No	263	96.0%	90	96.8%	86	96.6%	87	94.6%	
Yes	11	4.0%	3	3.2%	3	3.4%	5	5.4%	
History of Stroke									0.231
No	268	97.8%	89	95.7%	88	98.9%	91	98.9%	
Yes	6	2.2%	4	4.3%	1	1.1%	1	1.1%	

*Note:* Categorical variables are shown as *n* (%). *p*‐values were calculated based on log‐transformed variables. Different letters indicate statistically significant differences (*p* < 0.05) between groups, according to post‐hoc Bonferroni's test.

Abbreviations: AMI = acute myocardial infarction, DM2 = type 2 diabetes mellitus, IQR = interquartile range, SD = standard deviation, TSH = thyroid‐stimulating hormone, T4 = thyroxine.

**Figure 3:**
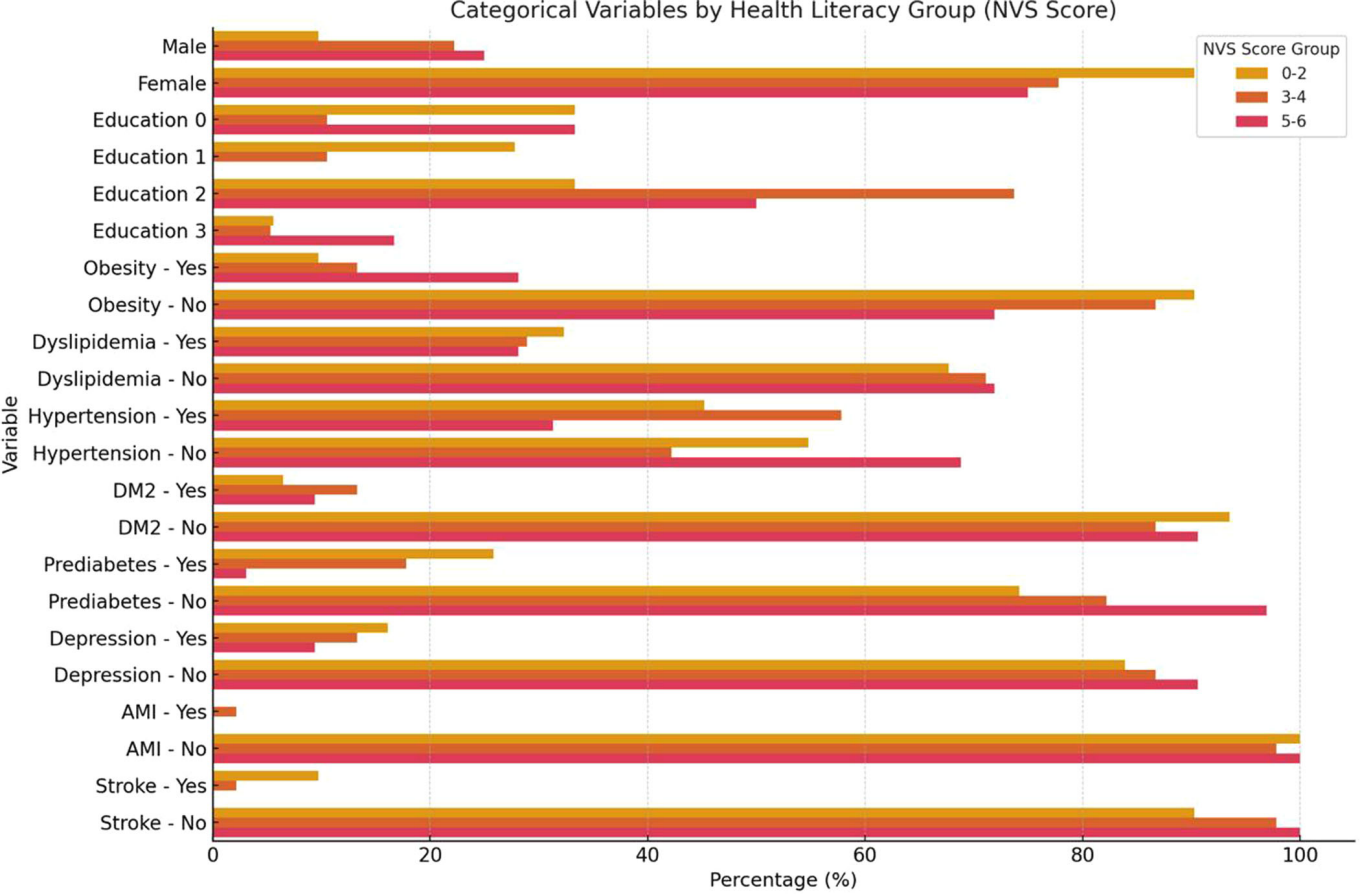
Distribution of categorical clinical variables across health literacy levels according to Newest Vital Sign (NVS) scores. Values are presented as percentages. AMI = acute myocardial infarction, DM2 = type 2 diabetes mellitus, NVS = Newest Vital Sign, TSH = thyroid‐stimulating hormone, T4 = thyroxine.

When comparing biochemical parameters across health literacy strata, median TSH values were significantly higher in the lowest literacy group (0–2) compared with those scoring 3–4 (*p* = 0.004) and 5–6 (*p* < 0.001). Patients who scored 3–4 had higher TSH values than those in the highest group (5–6) (*p* < 0.001). Patients in the lowest literacy group presented higher levothyroxine dose per kg than their counterparts in the middle group (*p* = 0.011), whereas FT4 did not differ significantly between groups (Table 1, Figure 2). With respect to comorbidities, clinical variables—including dyslipidaemia, hypertension, obesity, depression, prediabetes, DM2, previous myocardial infarction, and history of stroke—showed no statistically significant differences between literacy levels (all *p* > 0.05, Figure 3).

In multivariable regression analyses, higher health literacy was independently associated with lower serum TSH concentrations (3–4 group: β = −0.399, 95% CI −0.649; −0.148, *p* = 0.002; 5–6 group: β = −0.897, 95% CI −1.153; −0.641, *p* < 0.001) after adjustment for age, sex, duration of diagnosis, obesity, and comorbidities (dyslipidaemia, diabetes, hypertension). The association of health literacy with FT4 was marginal, showing a trend for a positive association in the fully adjusted model (β = 0.071, *p* = 0.063 and β = 0.061, *p* = 0.053 for 3–4 and 5–6 groups, respectively). A negative and significant association was observed between NVS score and levothyroxine dose per kg (3–4 group: β = −0.083, 95% CI −0.144; −0.022, *p* = 0.008; 5–6 group: β = −0.064, 95% CI −0.124; −0.004, *p* = 0.038). Results are summarised in Table 2.

**Table 2:** Association between health literacy levels and lnTSH, lnFT4, and levothyroxine dose (µg/kg/day).

	Univariate				Adjusted model			
lnTSH	B	95% CI		*p* value	B	95% CI		*p* value
Health Literacy								
0–2	Ref.				Ref.			
3–4	−0.389	−0.652	−0.126	0.004	−0.399	−0.649	−0.148	0.002
5–6	−0.883	−1.146	−0.620	< 0.001	−0.897	−1.153	−0.641	< 0.001
lnT4								
Health Literacy								
0–2	Ref.				Ref.			
3–4	0.064	−0.01	0.139	0.091	0.071	−0.004	0.145	0.063
5–6	0.053	−0.008	0.115	0.089	0.061	−0.001	0.122	0.053
Levothyroxine Dose								
Health Literacy								
0–2	Ref.				Ref.			
3–4	−0.094	−0.161	−0.028	0.006	−0.083	−0.144	−0.022	0.008
5–6	−0.067	−0.131	−0.004	0.038	−0.064	−0.124	−0.004	0.038

*Note:* The adjusted models for lnTSH and lnFT4 included age, sex, duration of diagnosis, dyslipidaemia, diabetes, hypertension, and obesity. The adjusted model for levothyroxine dose included age, sex, duration of diagnosis, dyslipidaemia, diabetes, and hypertension.

## 4 Discussion

In this study, we found a clear and consistent relationship between health literacy and biochemical control in patients with primary hypothyroidism. Individuals with higher Newest Vital Sign (NVS) scores had lower serum TSH concentrations, and this association remained significant even after adjusting for age, sex, disease duration, and major comorbidities. Each step up in literacy appeared to move patients closer to guideline‐recommended TSH targets, highlighting how functional literacy plays a direct role in treatment success [14, 15].

This effect cannot be explained simply by years of schooling. Although education and health literacy are related, they capture different abilities. The NVS evaluates skills such as reading comprehension and numeracy, which are essential for applying medical instructions in daily life and are not always reflected by formal education. In countries like Brazil, where educational quality is uneven, this distinction becomes especially important. Our findings suggest that patient evaluation should consider functional literacy, not only reported schooling [6, 7].

We also observed that literacy levels were not associated with differences in age, sex, formal education, or the prevalence of obesity, dyslipidaemia, hypertension, diabetes, or cardiovascular disease. This strengthens the interpretation that the relationship between literacy and TSH control reflects literacy‐specific mechanisms rather than baseline clinical profile [8, 16].

Adherence to levothyroxine is the most likely explanation for the association we found. The medication requires strict routines: fasting intake, avoiding interactions with food or other drugs, and taking it at the same time each day. Patients with limited literacy may struggle with these requirements, which can lead to TSH fluctuations. We did not measure adherence directly, but prior studies show that understanding instructions, managing missed doses, and recognising interactions strongly relate to literacy levels. Adherence is probably a key mediator, though other elements—such as communication with health professionals or general self‐care skills—may also play a role [14, 16, 17].

Another relevant finding was that patients with lower literacy required higher doses of levothyroxine per kilogram. This supports the idea that irregular intake or absorption issues could contribute to biochemical instability. On the other hand, associations with FT4 were weaker and lost significance after adjustment, although a marginal trend remained. This may be explained by the greater biological variability of FT4 compared with TSH, as well as transient influences such as dietary iodine, gastrointestinal absorption, acute illness, or the use of supplements like calcium or iron. Some patients may also take levothyroxine right before blood collection, which can artificially increase FT4 levels [18].

From a clinical point of view, these findings create opportunities for simple and low‐cost strategies to improve treatment outcomes. At our clinic, we are developing educational materials in plain language to help patients understand levothyroxine use, focusing on key issues such as fasting, missed doses, and timing. These actions align with the principles of the Brazilian public health system (SUS), which values equity, access, and patient empowerment [18, 19].

This study has limitations. First, its cross‐sectional design does not allow us to establish causality. Second, health literacy was assessed using the NVS, which, although validated, measures general literacy skills rather than thyroid‐specific self‐care behaviours [20]. Third, we did not evaluate medication adherence directly, which limits our ability to confirm its mediating role. Fourth, although all blood samples for TSH were collected in the morning to reduce circadian effects, some residual variability is possible. Finally, although 355 patients were initially identified, 81 were excluded according to predefined criteria, leaving 274 in the final analysis. Even with a smaller‐than‐expected sample, the associations remained consistent and biologically plausible.

Digital tools may offer additional ways to support patients with low literacy, including voice reminders, simplified mobile apps, and pictorial instructions. Future studies should test whether tailoring these interventions to patients' literacy levels can improve adherence and biochemical control [12, 19, 21].

Addressing health literacy is a practical and cost‐effective approach to strengthening endocrine care, particularly in settings with limited resources.

## 5 Conclusion

In summary, limited health literacy, as measured by lower NVS scores, was independently associated with higher serum TSH in patients with primary hypothyroidism. Individuals with greater literacy not only achieved better biochemical control but also required lower doses of levothyroxine per kilogram, suggesting more efficient treatment management. By contrast, associations with FT4 were weaker and did not remain significant after adjustment. These findings position health literacy as a modifiable determinant of treatment success. Simple strategies such as plain‐language counselling, teach‐back techniques, and visual aids could help patients achieve more stable thyroid control, with potential benefits in long‐term morbidity and quality of life. Future research should explore how literacy‐sensitive approaches can be integrated into routine thyroid care at scale.

## Author Contributions

**Jessyka Krause Meneses:** conceptualisation (lead), data curation (lead), writing – original draft (lead). **Daniella Araujo Muniz:** writing – review and editing (equal). **Débora Moroto:** writing – review and editing (equal). **João Roberto Maciel Martins:** writing – review and editing (equal). **Carolina Castro Porto Silva Janovsky:** conceptualisation (lead), supervision (lead), Project administration (lead), writing – review and editing (lead).

## Conflicts of Interest

Jessyka Krause Meneses is currently employed by Merck. However, this employment commenced after the study was designed, conducted, and completed. The other authors declare no conflicts of interest.

## Data Availability

Deidentified participant data and the data dictionary that support the findings of this study are available from the corresponding author upon reasonable request. The study protocol, statistical analysis plan, and informed consent form will also be made available upon request, for researchers whose proposed use of the data has been approved. During the preparation of this study, the authors used ChatGPT (OpenAI) to assist with language clarity and English readability. After using this tool, the authors carefully reviewed and edited the content to ensure accuracy and integrity and take full responsibility for the final content of this publication. The data collected and analyzed for this study will be made available upon reasonable request to the corresponding author. Deidentified participant data, data dictionary, and related documents such as the study protocol and statistical analysis plan will be shared. Data will be available with publication and can be requested via email to the corresponding author. Access will be granted upon approval of a proposal and signing of a data access agreement.
